# Effects of Oxygen and Carbon on Plants

**Published:** 1873-10

**Authors:** 


### EFFECTS OF OXYGEN AND CARBON ON PLANTS.

Professor Joseph Bohm has communicated to the Academy of Sciences, of Vienna, some curious and interesting observations in vegetable physiology. He has found that young plants produced from seeds germinating in pure oxygen gas of ordinary density speedily die, although they continue to consume oxygen to as great an extent as when they are growing in the atmospheric air. The young plants thrive, however, in pure oxygen when the density of the latter is reduced so as to represent only a pressure of about six inches of mercury, or when pure oxygen of ordinary density is mixed with four-fifths of its volume of hydrogen. Professor Bohm also investigated the action of carbon upon the growth and greenness of plants, and found that an intermixture of only two per cent. of carbonic acid in the air in which plants are growing suffices to retard the formation of green coloring matter (chlorophyl), and that the process is almost or entirely suppressed in an atmosphere containing twenty per cent. of this gas. No germination of seeds took place in an atmosphere consisting of one-half carbonic acid. From his experiments the professor concludes that either the atmosphere of our planet was much richer in carbonic acid than at present in early geological periods, especially during the formation of coal deposits, or the plants of those periods, in their relation to carbonic acid, must have been very differently constituted from their existing descendants.—*Med. and Sur. Reporter.*

